# Population epigenetics: Historical notes and applications in human health

**DOI:** 10.1590/1678-4685-GMB-2025-0203

**Published:** 2026-05-01

**Authors:** Sergio Russo Matioli

**Affiliations:** 1 Universidade de São Paulo, Instituto de Biociências, Departamento de Genética e Biologia Evolutiva, São Paulo, SP, Brazil.

**Keywords:** Epigenetic inheritance, population genetics, Lamarckism, population epigenetics

## Abstract

A key factor contributing to the success of Darwin and Wallace’s theory of biological evolution by natural selection was its population-level perspective. This conceptual framework was not immediately adopted, largely due to the enduring intuitive appeal of Lamarckian ideas. The development of genetics during the twentieth century provided compelling evidence that effectively excluded Lamarckian mechanisms from the mainstream understanding of evolutionary processes. Some naturalists, however, proposed mechanisms by which environmental factors could influence genotypes in shaping phenotypes, later attributed to chemical modifications of DNA or chromosomal proteins, among others. The field of population epigenetics emerged with the aim of extending the well-established discipline of population genetics by incorporating such phenomena. This review seeks to provide a historical background on this subject and to examine how advances in both contemporary epigenetics and population epigenetics have been achieved, as well as their implications for the study of human diseases, particularly regarding their contribution to the phenomenon of missing heritability. Because there are major taxonomic differences in the transgenerational inheritance of epigenetic modifications, the potential effects of epigenetic architecture on phenotypes of interest also differ, as in the case of mammals and, in particular, humans.

## Historical notes

### Evolution and population genetics

Although the concept of biological evolution can be traced back to ancient philosophers, its most significant impetus came from the work of Charles Darwin and Alfred Russel Wallace (Darwin, 1859; Wallace, 1858) Their theory of evolution by natural selection was widely shared in both scientific and public circles soon after the publication of the Origin of species (Mayr, 1982; Bowler, 2009) A distinctive and original feature of the theory of evolution by natural selection is its fundamentally population-based perspective. In this framework, natural selection operates entirely based on heritable variation among individuals within natural populations. By contrast, the alternative evolutionary theory proposed by Jean-Baptiste Pierre Antoine de Monet, Chevalier de Lamarck (Lamarck, 1809), emphasized the adaptive responses of individual organisms to environmental challenges and asserted that such acquired characteristics could be inherited by subsequent generations. These processes were not based on differential survival or reproduction and, therefore, did not depend on population-level variation. Despite its initial impact on biology and on science in general, the theory of evolution by natural selection was gradually abandoned in favor of Lamarckian and other alternative ideas during the final decades of the 19th century and even into the early decades of the 20th century (Bowler, 1983) This period became known as “the eclipse of Darwinism,” a term later popularized by Julian Huxley (Huxley, 1942)  In the early twentieth century, with the growing awareness of Mendelian inheritance, the genetic substrate of natural selection eventually assumed a more substantial form, though not without intense debate. On one side were the Mendelians, scientists who adhered to a particulate view of genetic variability. On the other side were the biometricians, scientists who considered continuous variation of traits to be the main substrate of natural selection. This conflict was theoretically resolved by Ronald Aylmer Fisher in 1918, through his seminal paper in which he proposed that the continuous variation of traits could be explained by particulate inheritance (Fisher, 1918). This resolution can be considered to have a dialectical structure, producing a synthesis that became the foundation for later research on genetics and evolution of every trait. 

The field of population genetics is often dated to 1908, when Godfrey Harold Hardy and Wilhelm Weinberg independently published papers on the population properties of genes and genotypes under Mendelian inheritance (Hardy, 1908; Weinberg, 1908; Edwards, 2008) Their work were later referred to as the Hardy-Weinberg principle. In the first decades of the twentieth century, the field was further developed through major contributions from Fisher (1930), Haldane (1924, 1927, 1932), and Wright (1931, 1932, 1938, 1943) The work of these and other authors established what later came to be called the classical period of population genetics, during which natural populations were thought to harbor little genetic variation. This view changed dramatically in 1966, when analyses of allozyme polymorphisms in natural populations revealed unexpectedly high levels of heterozygosity (Hubby and Lewontin, 1966; Lewontin and Hubby, 1966) During the classical period, polymorphisms were believed to be maintained by balancing selection (e.g., heterozygote advantage) or were considered transient states leading to allele substitution. These empirical observations, together with the proposal that protein sequences evolve in a clockwise fashion (Zuckerkandl and Pauling, 1962, 1965; see also Morgan, 1998), lead to the development of the neutral theory of molecular evolution, formulated mainly by the Japanese biologist Motoo Kimura and his coworkers (Kimura and Crow, 1964; Kimura, 1968; Kimura, 1969; King and Jukes, 1969; Ohta, 1973) According to this theory, most of variation at the protein level is neutral or almost neutral with respect to being subject of natural selection. In the following decades, technological developments in the analyses of DNA polymorphisms permitted surveys of genetic variability of natural populations, crops, livestock and human samples as well. Those techniques were RFLP (restriction fragment length polymorphism), RAPD (random amplified polymorphisms of DNA), microsatellites (regions of short tandem repeats of DNA sequences), and SNPs (single nucleotide polymorphisms), among others. These improvements on the assessment of genetic variability have been reviewed by Grover and Sharma (2016) : as a general result, the empirical levels of genetic variability were even more pronounced than in protein-based evaluations. 

### Molecular basis of human genetic diseases

**Figure 1-:**
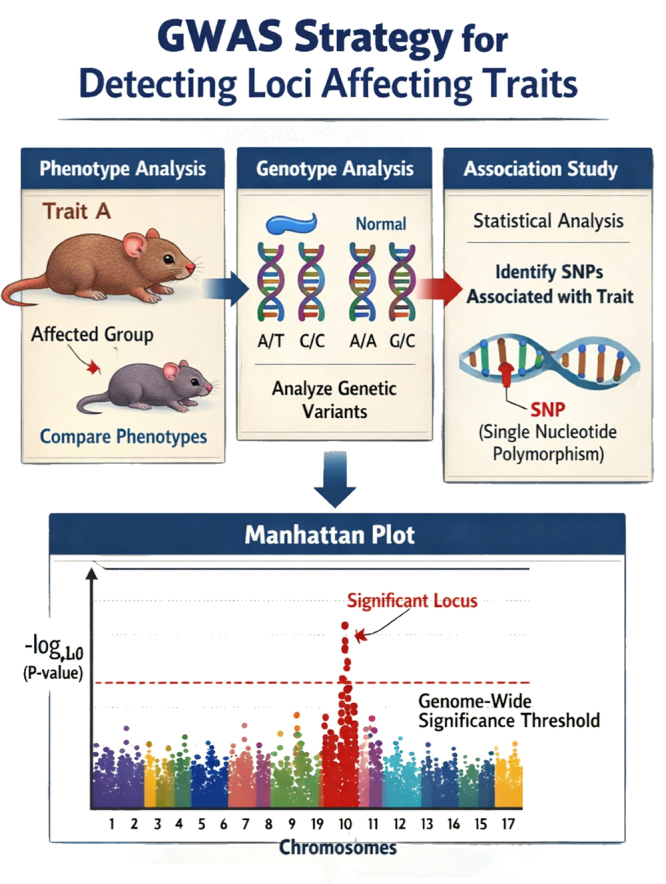
Diagram showing the GWAS approach used to localize, in the genome, regions in linkage disequilibrium with variants that can affect the trait under study. Although the phenotypes shown are in mice (with an arbitrary number of chromosomes), this approach is widely used to analyze human samples.

Before the DNA was found to be the molecule linked to the genetic material, genes were generally considered as abstract factors. Sir Archibald Edward Garrod, an English physician, hypothesized that genes acted in metabolism causing heritable diseases (Garrod, 1902, 1931) Later, Beadle and Tatum (1941) proposed the one gene - one enzyme hypothesis. Protein polymorphisms linked to human diseases started to be studied by the pioneer work of Pauling et al. (1949) , when they showed that sickle cell anemia was due to differences in hemoglobin sequences that were detectable by gel electrophoresis. The DNA markers, as soon they become available, were also widely used to study human genetic diseases with complex inheritance (hereafter called by its most common name, “complex diseases”) For an historical review of uses of molecular markers, see Ku et al. (2010) The widespread use of DNA markers enabled the development of QTL (quantitative trait loci) studies in crops, livestock, and experimental organisms, and GWAS (genome-wide association studies, Figure 1) in humans. The main distinction between these approaches concerns ethical constraints: it is not ethically admissible to subject human individuals to inbreeding or to direct mating based on phenotypic traits. In contrast, such procedures are permitted in experimental or commercial organisms, provided they adhere to current ethical guidelines. Beyond these ethical considerations, the QTL and GWAS approaches differ in terms of biological implications. QTL studies, particularly those involving strongly inbred lineages, often rely on genetic variability that does not reflect natural population structure. In extreme cases, such as in mice or crop lines that have undergone many generations of inbreeding, only two genome-wide homozygous backgrounds are indeed being analyzed (Jahuey-Martínez et al., 2024; Hassanine et al., 2025)

The overall results of GWAS on human diseases have led to the phenomenon commonly known as “missing heritability.” This term was first used by Maher (2008) , who argued that even with large samples and dense genomic coverage, the predictive power of observed genotypes for human height, long known to be highly heritable (≈70-80%), was much lower than expected. After the phenomenon of missing heritability was established, some propositions of possible causes for it were put forward. From the population genetics point of view, the hypothesis concerning the occurrence or rare alleles was one possibility. This hypothesis was considered because the cutoff frequency of markers was high and because the alleles that causes the most pronounced phenotypes tended to be eliminated by natural selection. Another hypothesis concerns the presence of structural variants: insertions, deletions or inversions, copy number variation here included. Epistasis can inflate previous estimates of heritability and poses some problems in detecting combined effects of more than a single locus. Environmental and epigenetic causes can also contribute to increase the estimates of heritability, and, at this time, epigenetic effects were not widely considered in the analyses (see Manolio et al., 2009) 

### Epigenesis and epigenetics

**Figure 2-:**
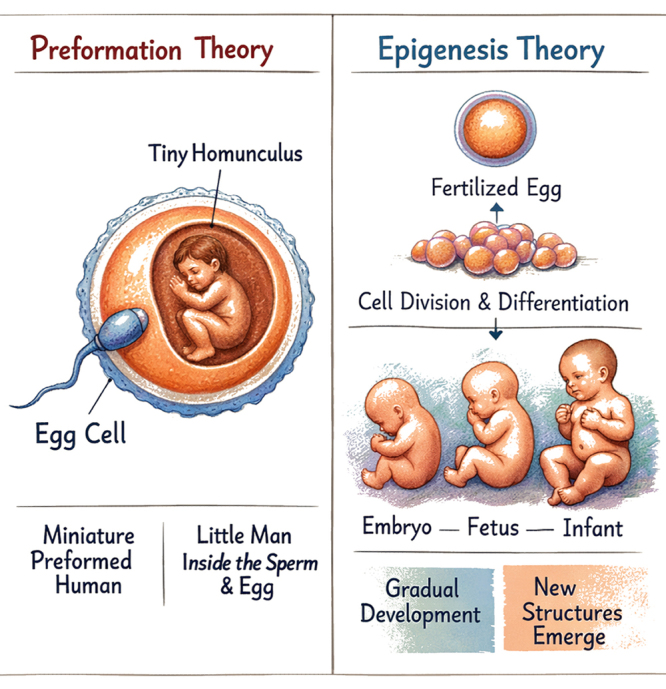
Illustration showing the differences between the preformation and epigenesis theories of embryonic development.

The word *epigenesis* refers to an embryological theory of development that can be traced back to classical antiquity. Although Aristotle did not explicitly employ the term, he articulated a related idea. In *On the Generation of Animals* (Platt, 1912), Aristotle holds that development proceeds through an active principle in the semen acting upon the material provided by the egg. The theory of epigenesis stood in opposition to preformation theory, which, in its more radical forms, held that the egg already contained a miniature organism that would only grow during development (Figure 2). Attributing to Aristotle the origins of the epigenesis/preformation debate has been disputed (Goy, 2018), but there is broad agreement that the term *epigenesis* itself was introduced by the English physician William Harvey in 1651 (Harvey, 1651; see also Casetta, 2020; Eriksen, 2022) Harvey contrasted epigenesis, progressive, part-by-part formation, with metamorphosis, a reorganization of an already formed organism. Marcello Malpighi, an Italian physician commonly regarded as a proponent of preformation theory (Malpighi, 1672), made important contributions to embryology by using a compound microscope to follow the development of the chick embryo and to study insect anatomy. Caspar Friedrich Wolff, a German medical student, published his undergraduate thesis *Theoria Generationis* in 1759 (later reprinted in 1774; Wolff, 1774 translated by Aulie, 1961), in which he presented his studies on the development of vegetal structures, such as flowers, and on the formation of organs in chicken embryos. This work, along with his subsequent studies, is considered the foundation of modern embryology and a revival of the concept of epigenesis. Karl Ernst von Baer, an Estonian naturalist, after conducting comparative studies on animal embryology, despite being a critic of biological evolution, specially to Darwin’s ideas (Brauckmann, 2008) proposed several laws that potentially linked embryology to evolution. For example, his first law: “the more general characters of a broader group appear earlier in the embryo than the more special characters” (von Baer, 1828, trans. Henfrey and Huxley, 1853) The von Baer laws later inspired Haeckel’s “biogenetic law” which stated that “ontogeny recapitulates phylogeny”, under the assumption of biological evolution (Haeckel, 1866; see also Haeckel, 1880).

The word “epigenetics” was coined by the British embryologist Conrad Hal Waddington (Waddington, 1942)  to mean the genetic basis of developmental processes that can be implied in phenotypical variation. It was long ago known that organisms present phenotypic plasticity, that is, the phenotype presented by individuals depends on the environment they have developed, and the modifications permit the individuals to deal better with the environmental challenges. This was present as the use and disuse law of Lamarck. In 1953, Waddington described the phenomenon of what he called “genetic assimilation”: he studied the effects of selection for a trait of *Drosophila melanogaster* that presented phenotypic plasticity (*crossveinless*, influenced by heat shock), and the selected lineages started to display the *crossveinless* phenotype constitutively, i.e. even without being exposed to the heat shock (Waddington, 1953). Waddington also described what is called “genetic canalization”. He called a canalized phenotype the phenotype that remains unchanged even with the presence of mutations that were expected to change this phenotype (Waddington, 1957, Figure 3). At the same period, the German/American geneticist Richard Benedict Goldschmidt published a controversial book, “Material basis of evolution” (Goldschmidt, 1940) In this book, Goldschmidt proposes that there are two distinct levels of biological evolution: The microevolution occurs within populations and the processes responsible for it are those traditionally studied, up to the date, under the population genetic theory. However, Goldschmidt postulated that great leaps of evolution are due to “macromutations”, that could be caused by great rearrangements of the genetic material, as in chromosomal mutations. Some experiments done within Goldschmidt’s lab were related to the production of phenocopies of *Drosophila* species, a word coined by him in 1935 (Goldschmidt, 1935, Goldschmidt and Piternick, 1956, 1957a, b) Phenocopies refer to phenotypes that are like those produced by genetic mutants but that were provoked by environmental stimuli, as heat shock, chemical induction or radiation. It is important to note that phenocopies are due to phenotypic plasticity and it does not imply heritable epigenetic change.

**Figure 3-:**
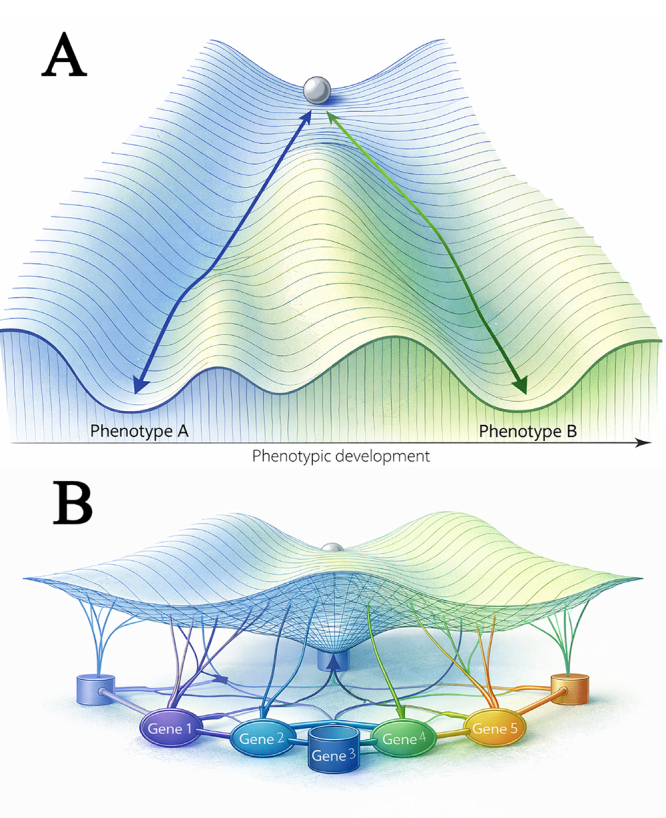
(A) The developmental landscape as envisioned by Waddington (1957). According to his view, the phenotype is canalized during evolution in a way that makes it more robust to environmental or genetic perturbations. (B) The landscape resulting from the action of genes that can interact with one another. Based on his original figures.

In the mid-20th century, there were initiatives interpreted as attempts to shield the central axis of evolutionary scholarship from what were considered remnants of Lamarck’s ideas. Historians of science debate whether these initiatives were coordinated or whether they simply followed the prevailing scientific consensus of the period. The foundation of the Society for the Study of Evolution in 1946, and the launch of its scientific journal in 1947, are interpreted within the framework of such a coordinated effort (Smocovitis, 1994) Meanwhile, in the USSR, the practice of so-called Lysenkoism, a doctrine based on Lamarckian views and applied to crop production, was promoted. This doctrine, advanced by the agronomist Trofim Lysenko during the Cold War, was adopted in the Soviet Union because it was regarded as more theoretically compatible with socialism than Darwin’s concept of the “struggle for existence”. By contrast, the opposite view applied to politics, known as social Darwinism and disseminated by the English polymath Herbert Spencer, held that welfare could be achieved without interference, by allowing individuals to find their own paths in society. Both Lysenkoism and social Darwinism produced catastrophic outcomes, and this can be regarded as a lesson in the dangers of extrapolating scientific views into political practice (Borinskaya et al., 2019; Davis, 2017; Reilly, 2015; Marwah, 2024)

**Figure 4 -:**
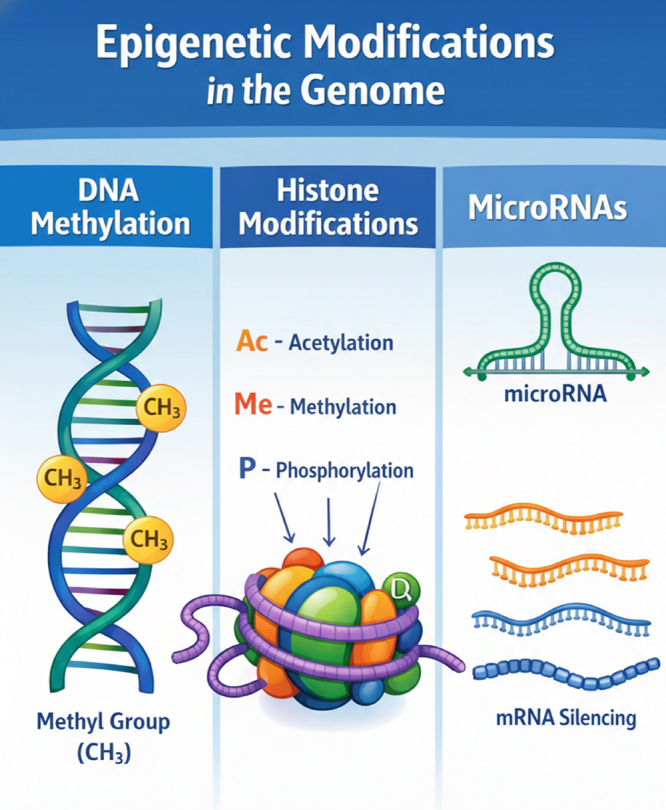
Three examples of epigenetic modifications that can affect phenotypic manifestations: chemical modifications of DNA nitrogenous bases, chemical modifications of chromatin proteins such as histones, and the action of small RNAs that can interact with DNA molecules.

In 1956, the Canadian geneticist Royal Alexander Brink described, in maize, the phenomenon of paramutation, by which the heritable expression of a gene is altered by allele of another gene, without modification in the gene itself. (Brink, 1956). This property was later attributed to *cis-trans* heritable regulatory interaction with small-RNA/chromatin involvement in maize (Deans et al., 2024). In 1971 the English geneticist Mary Frances Lyon proposed that one possible mechanism of inactivation of the X chromosome should be by methylation of the cytosine bases (Lyon, 1971). Methyl cytosine was first identified as a constituent of nucleic acids in 1925 by Johnson and Coghill (Johnson and Coghill, 1925) , found to be present in mammalian DNA from some tissues in 1962 (Doskocil and Sorm (1962) , and, after that, postulated to alt gene activity during development in many organisms (review by Holliday and Pugh, 1975). In 1989, Jablonka and Lamb published an article that suggested that chemical modifications in genetic material can be viewed as a Lamarckian inheritance of acquired traits (Jablonka and Lamb, 1989). In late 1980s, it was demonstrated that the same chromosomal region was involved in both Angelman and Prader-Willi human syndromes (Magenis et al., 1987, Knoll et al., 1989). It was also demonstrated that phenotypic differences between these syndromes were due to its genitor origin, therefore with differential imprinting (Nicholls et al., 1989). In 1991, the widespread differential expression of maternal and paternal genes in mammals were reported (Barlow *et al*., 1991; Bartolomei et al., 1991 and DeChiara et al., 1991). In 2000, it was demonstrated that there was a general reprogramming of methylation marks of the paternal genome in early embryos of mammals. (Mayer et al., 2000; Oswald et al., 2000). Besides modification of genes without altering their sequences by methylation, it has been noticed that small RNAs and chromatin proteins alterations can also cause epigenetic modifications. The methylation marks in DNA, chemical modifications on histones, and the role of small RNAs are represented in Figure 4. Recent reviews on genomic imprinting and its persistence after reprogramming in mammals can be found in Hubert and Demars (2022)  and Inoue (2023) .

### Population epigenetics

The first use of “population epigenetics” as the name of a research field was by Edwards (2008) , but Greally (2017)  attributes the first concepts related to this field to Keller (1995). In that work, although aware that one type of epigenetic phenomenon is related to DNA methylation, Keller focused on phenomena involving autoregulatory transcription factors, using mathematical modeling to study the stability of these alterations. However, some authors do not consider Keller’s work to fall within the domain of population epigenetics, since transcription factors are viewed as mediators that modify methylation patterns rather than being epigenetic marks themselves (e.g., Ptashne, 2007, 2013; Henikoff and Greally, 2016) . As in the field of population genetics, population epigenetics relies on mathematical models intended to predict population-level outcomes based on variable parameters. For example, in the context of population genetics, it is known that spontaneous mutation rates in the human genome are very low, approximately 1.2 × 10⁻⁸ per nucleotide per generation (Kong et al., 2012), and that 98 to 206 de novo mutations occur per transmission event, including structural variants (Porubsky *et al*., 2025). These low values are consistent with the expected occurrence of low-frequency polymorphisms for deleterious genes under models of mutation-selection balance (e.g., Weghorn et al., 2019). Traditional population genetics deals with mutants (rare alleles), polymorphisms (more common alleles, typically with frequencies around 0.01), and variants (a term more often used in human genetics). Population epigenetics, on the other hand, aims to incorporate epigenetic phenomena at the population level by introducing the concept of epialleles, analogous to classical genetic alleles but capable of changing during an individual’s lifetime without altering the nucleotide sequence. The term epialleles was proposed by Jacobsen and Meyerowitz (1997) , who initially used the expression “epi-alleles” to describe heritable properties at the SUPERMAN locus in *Arabidopsis thaliana*. The term metastable epialleles was later introduced by Rakyan et al. (2002)  to characterize genes subject to imprinting and embryonic epigenetic reprogramming in mammals. Keller (1995) studied populations exhibiting two epigenetic states under different selection regimes and mutation rates between states. When mutation rates were very low (i.e., when epigenetic states were stable), population responses to selection resembled the classical population genetics model of a single locus with two alleles. Geoghegan and Spencer (2012)  analyzed populations containing two or more epialleles under varying levels of selection. Again, population equilibria depended on the intergenerational stability of the epialleles. Empirical determination of DNA methylation status was historically based on differential enzymatic digestion using methylation-sensitive and -insensitive enzymes, a laborious process until 1992, when Frommer et al. (1992)  developed a genomic sequencing method that preserved methylation information. Husby (2022) has reviewed the literature about studies that performed analyses of methylation profiles in DNA samples of natural populations. In those studies that related these patterns with phenotypic traits, most of them were correlational (see also Chapelle and Silvestre, 2022).

## Applications in human health

### Population epigenetics and human genetic epidemiology

Since the first studies with mathematical models of populational epigenetics, it becomes clear that the most relevant factor concerning the importance of epigenetic marks at the populational level is the transgenerational stability of the markers. As in other mammals, there is a reprogramming of epigenetically modified genetic material in early phases of the human embryos. This occurs also in the germline. However, not all epigenetic marks are erased, the imprinted genes are not reprogrammed (Daxinger and Whitelaw, 2012 for a review of epigenetic markers comprising methylated nucleotides, modified histones and small RNAs). Slatkin (2009)  studied mathematically the effect of recurrence risk of traits that are subject of epigenetic effects, because of its importance in the issue of the missing heritability. He also concluded that this is also dependent on the transgenerational stability of the epigenetic marks.

One of the classical examples of an intergenerational effect that have been attributed to epigenetic causes is related to the case of the Dutch Hunger Winter (Nederlands 1944-1945) and the severe health consequences on the adults that were born during this period (Barker *et al*., 1989, for example). Later it was shown that, even six decades after, the imprinted IGF2 gene was found to be less methylated in people born within these years when compared to unexposed counterparts (Heijmans et al., 2008).

The significance of epialleles in population epigenetics can be more clearly understood by considering two limiting scenarios with respect to the transgenerational stability of epigenetic marks. When stability is very high, the behavior of population parameters converges with expectations under the classical framework of population genetics. In this context, a stably inherited epigenetic modification such as cytosine methylation can be treated conceptually as an additional heritable state, akin to a ‘fifth nucleotide’ within the DNA sequence (Richards, 2006; Johannes et al., 2009). Conversely, when stability is low, the effect at the population level manifests as an increased risk of recurrence of particular traits, a phenomenon analogous to the influence of shared environmental conditions (Slatkin, 2009). The outcomes observed in intermediate cases would, accordingly, be intermediate between these two extremes of transgenerational stability (Daxinger and Whitelaw, 2012).

Despite the current availability of millions of genetic markers and even complete genome sequences for individuals, our ability to characterize environmental exposures with comparable resolution remains limited. This imbalance between the depth of molecular-level genomic data and the scarcity of high-quality environmental information continues to hinder our understanding of phenotype determination. Whereas genome sequencing routinely generates terabytes of data that can be systematically catalogued (1000 Genomes Project Consortium, 2015), the environmental exposures to which an individual has been subjected are often heterogeneous, transient, and difficult to quantify with precision (Wild, 2005; Rappaport and Smith, 2010) This disparity underscores the urgent need for integrative approaches, such as exposomics and longitudinal monitoring, that aim to capture the complex interactions among genotype, environment, and phenotype (Patel and Manrai, 2015; Vermeulen et al., 2020)

**Figure 5-:**
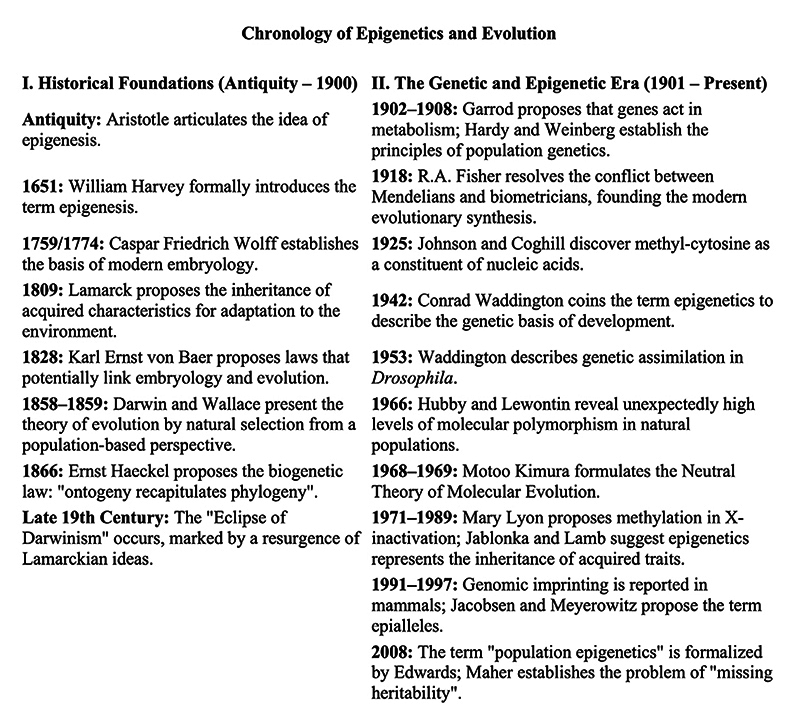
Chronology of some of the key events related to the fields of epigenesis, population genetics, and population epigenetics. Details of these and other contributions, as well as citations, can be found in the main text.

Some key events on the development of epigenesis, population genetics, epigenetics and population epigenetics are represented in Figure 5.

## Data Availability

All data used in this review came from bibliographical sources listed in References and Internet resources sections and are available to the readers depending on the permissions granted to them.
